# The anti-HIV drug abacavir stimulates β-catenin activity in osteoblast lineage cells

**DOI:** 10.1093/jbmrpl/ziae037

**Published:** 2024-03-19

**Authors:** Arnold Z Olali, Jennillee Wallace, Hemil Gonzalez, Kelsey A Carpenter, Niyati Patel, Lee C Winchester, Anthony T Podany, Ishwarya Venkatesh, Srinivas D Narasipura, Lena Al-Harthi, Ryan D Ross

**Affiliations:** Department of Anatomy & Cell Biology, Rush University Medical Center, Chicago, IL 60612, United States; Department of Microbial Pathogens and Immunity, Rush University Medical Center, Chicago, Illinois 60612, United States; Department of Microbial Pathogens and Immunity, Rush University Medical Center, Chicago, Illinois 60612, United States; Department of Microbial Pathogens and Immunity, Rush University Medical Center, Chicago, Illinois 60612, United States; Division of Infectious Diseases, Department of Internal Medicine, Rush University Medical Center, Chicago, IL 60612, United States; Department of Anatomy & Cell Biology, Rush University Medical Center, Chicago, IL 60612, United States; Department of Anatomy & Cell Biology, Rush University Medical Center, Chicago, IL 60612, United States; UNMC Center for Drug Discovery, University of Nebraska Medical Center, Omaha, NE 68198, United States; UNMC Center for Drug Discovery, University of Nebraska Medical Center, Omaha, NE 68198, United States; Department of Internal Medicine, Drug Discovery Center, Rush University Medical Center, Chicago, IL 60612, United States; Department of Microbial Pathogens and Immunity, Rush University Medical Center, Chicago, Illinois 60612, United States; Department of Microbial Pathogens and Immunity, Rush University Medical Center, Chicago, Illinois 60612, United States; Department of Anatomy & Cell Biology, Rush University Medical Center, Chicago, IL 60612, United States; Department of Microbial Pathogens and Immunity, Rush University Medical Center, Chicago, Illinois 60612, United States; Department of Orthopedic Surgery, Rush University Medical Center, Chicago, IL 60612, United States

**Keywords:** abacavir, Wnt/β-catenin, osteoblast, antiretroviral, BMD

## Abstract

Bone mineral density (BMD) loss in people living with HIV occurs with the initiation of combined antiretroviral therapy (cART), particularly with tenofovir disoproxil fumarate (TDF) containing cART. Switching from TDF to abacavir (ABC) or dolutegravir (DTG) leads to increased BMD. Whether BMD gains are due to cessation of TDF or anabolic effects of ABC or DTG is unclear. We investigated the effects of ABC and DTG on osteoblast lineage cells *in vitro* and *in vivo*. Primary human osteoblasts and male C57BL/6 mice were treated with individual antiretrovirals (ARVs) or a combination of ABC/DTG/lamivudine (3TC). Nearly all ARVs and cART inhibited osteogenic activity *in vitro*. Due to the importance of Wnt/β-catenin in bone formation, we further investigated ARV effects on the Wnt/β-catenin pathway. ABC, alone and as part of ABC/DTG/3TC, increased osteoblastic β-catenin activity as indicated by increased TOPFlash activity, hypo-phosphorylated (active) β-catenin staining, and β-catenin targeted gene expression. Mice treated with TDF had decreased lumbar spine BMD and trabecular connectivity density in the vertebrae, while those treated with ABC/DTG/3TC reduced cortical area and thickness in the femur. Mice treated with ABC alone had no bone structural changes, increased circulating levels of the bone formation marker, P1NP, and elevated expression of the Wnt/β-catenin target gene, Lef1, in osteocyte enriched samples. Further, bones from ARV-treated mice were isolated to evaluate ARV distribution. All ARVs were detected in the bone tissue, which was inclusive of bone marrow, but when bone marrow was removed, only TDF, ABC, and DTG were detected at ~0.1% of the circulating levels. Overall, our findings demonstrate that ABC activates Wnt/β-catenin signaling, but whether this leads to increased bone formation requires further study. Assessing the impact of ARVs on bone is critical to informing ARV selection and/or discovery of regimens that do not negatively impact the skeleton.

## Graphical Abstract

**Graphical Abstract:**
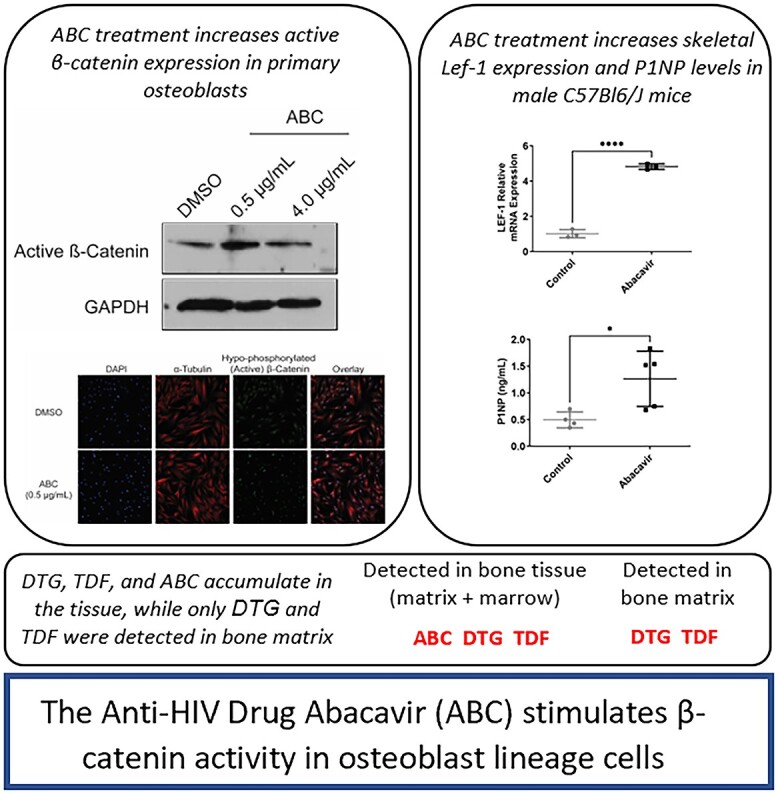

## Introduction

Combined antiretroviral therapy (cART) has significantly reduced HIV-associated deaths. However, cART-treated people living with HIV (PLWH) have an increased risk of osteoporosis,^(^^1^^)^ driven by a combination of traditional and HIV-related factors,^(^^2–4^^)^ including cART.^(^^5–8^^)^ While all cART regimens are associated with bone mineral density (BMD) loss, the magnitude is antiretroviral (ARV) dependent. Tenofovir disoproxil fumarate (TDF) is associated with 2-4% BMD loss within the first 2 years.^(^^9^^)^ Clinical data support avoiding TDF and using less bone-toxic ARVs in PLWH at risk for osteoporosis,^(^^1^^)^ like abacavir (ABC) or dolutegravir (DTG). PLWH switching from TDF-containing cART to ABC^(^^10^^,^^11^^)^ or DTG^(^^12^^)^ see BMD recovery. However, it is unclear whether this is due to cessation of TDF or positive effects of ABC and DTG. PLWH initiating ABC^(^^13–15^^)^ or DTG^(^^16^^)^ lose BMD, but this could be due to the rapid rise in CD4+ cells post cART-mediated immune reconstitution.^(^^17^^)^ ABC and DTG are not used in pre-exposure prophylaxis (PrEP), therefore their HIV-independent effects on bone are unknown. In this study, we investigated the effects of TDF, ABC, and DTG individually and as part of a standard clinical cART on primary human osteoblasts (HOBs). We also investigated TDF, ABC, and ABC/DTG/3TC effects on BMD *in vivo*.

Osteoblasts form bone matrix and influence bone resorption through the expression of RANKL, which induces osteoclast-mediated bone resorption.^(^^18^^)^ Wnt/β-catenin signaling is critical to osteoblast activity^(^^19^^)^ and HIV pathogenesis, suppressing viral replication.^(^^20^^,^^21^^)^ Previously we have shown increased expression of sclerostin, a Wnt antagonist, in cART treated women with HIV.^(^^22^^)^ Others noted increased circulating Dickkopff-1 (Dkk1), another Wnt antagonist in rats treated with ABC.^(^^23^^)^ Due to the importance of Wnt/β-catenin signaling in bone formation and the potential responsiveness to ARVs, we evaluated the impact of ARVs on Wnt/β-catenin signaling in osteoblasts.

## Materials and methods

### Antiretrovirals

Antiretrovirals were obtained from the NIH AIDS reagent program (now available at beiresources.org).

### Osteoblasts culture and differentiation

Primary HOBs from a 30-year-old Caucasian female donor (Promocell, Heidelberg, Germany) were cultured in collagen-coated 12-well plates (Corning) and assessed between passages 2 and 6. Culture media specifics are provided in the supplemental materials. *In vitro* ARV concentrations are chosen to represent the plasma ranges detected in PLWH. Tenofovir disoproxil fumarate concentrations were 0.01 and 0.64 μg/mL,^(^^24^^)^ abacavir (ABC) 0.5 and 4.0 μg/mL,^(^^25^^)^ dolutegravir (DTG) 0.02 and 0.48 μg/mL,^(^^26^^)^ and lamivudine (3TC) 0.15 and 2.4 μg/mL.^(^^27^^)^ The ABC/DTG/3TC combination treatment was prepared by combining the low doses of ABC, DTG, and 3TC (0.5 [ABC], 0.02 [DTG], 0.15 [3TC]) and the high doses (4.0 [ABC], 0.48 [DTG], 2.4 [3TC])) together.

### C57BL/6J mice

Animal studies were approved by the Rush University Institutional Animal Care and Use Committee. Male C57BL/6J mice were purchased from Jackson Laboratory (Bar Harbor, ME, USA). Mice were caged in groups of 5, maintained on a 12-hour dark/light cycle, and provided standard chow (2018, Teklad; 1% calcium, 0.7% phosphorus) and water *ad libitum.***Antiretrovirals** were mixed into the chow at clinical doses adjusted for the mouse metabolic rate according to FDA recommendations^(^^28^^)^ – 51 mg/kg TDF, 105 mg/kg ABC, or ABC/DTG/3TC cART at 105 mg/kg ABC, 8.7 mg/kg DTG, and 51 mg/kg 3TC, respectively. Control mice were provided standard rodent chow.

Three separate *in vivo* experiments were performed. All studies were initiated in 12-week old mice, a point where longitudinal bone growth has plateaued in the C57BL/6J mouse.^(^^29^^)^ The first evaluated oral administration of ARVs. We have previously measured biodistribution of orally administered ARVs in an HIV-infected mouse model, but skeletal accumulation was not evaluated.^(^^30^^)^ Mice received control chow, TDF, monotherapy, or ABC/DTG/3TC, for 4 weeks (*n* = 4, 7, 7 for control, TDF, and ABC/DTG/3TC, respectively). In the second experiment, mice received daily oral administration of control chow, TDF monotherapy, or ABC/DTG/3TC, cART for 6 weeks (*n* = 10 per group). In the third experiment, mice received either control chow or ABC monotherapy for 6 weeks (*n* = 5 per group). Detailed tissue collection methods are presented in the supplemental material.

### Cell viability

Human osteoblasts differentiated in collagen-coated 96-well plates with continuous exposure to various concentrations of TDF, ABC, DTG, or 3TC individually for 14 days with media changes every 3 days. Dimethyl sulfoxide (DMSO) was used as a vehicle control. Viability was assessed using the 3-(4,5-dimethylthiazol-2-yl)-2,5-diphenyl tetrazolium bromide (MTT) reagent (5 mg/mL, EMDmillipore, Bedford, MA, USA).

### Alizarin red staining and alkaline phosphatase activity

Human osteoblasts were differentiated in collagen-coated 12-well plates with continuous exposure to TDF, ABC, DTG, or 3TC individually or with ABC/DTG/3TC cART for 14 days with fresh media every 3 days. DMSO was used as a vehicle control. On day 14, cells were washed with PBS, fixed with formaldehyde and stained with alizarin red at pH 4.2 (Sigma-Aldrich). Bound alizarin red was extracted and quantified. Detailed methods are presented in the supplement. To assess alkaline phosphatase (ALP) activity, cells were washed with PBS and stained with a 1-step NBT/BCIP Substrate Solution (ThermoFisher Scientific). ALP staining intensity was quantified using Image J version 2.1 (NIH, Bethesda, MD, USA) and normalized to DMSO-treated HOBs.

### Quantitative real-time PCR

Human osteoblasts were differentiated for 13 days and then treated with ARVs for 24 hours. The expression of target genes was evaluated using qPCR (see Supplemental methods). Relative expression was calculated using the 2^dCT method with β-actin as the housekeeping gene. Primers sequences are presented in Supplementary Tables 1 and 2.

### Western blot

Human osteoblasts were differentiated for 13 days and treated with ARVs for 24 hours. Total protein content was measured using a bicinchoninic acid (BCA) assay (Bio-Rad, Des Plains, IL). Blotting was performed to assess total β-catenin (1:10 000, Sigma-Aldrich), hypo-phosphorylated (active) β-catenin (1:1000, USBiological), and glyceraldehyde 3-phosphate dehydrogenase (GAPDH, Sigma-Aldrich). Detailed methods are presented in the supplement.

### Immunofluorescence staining

Human osteoblasts were seeded on collagen-coated coverslips. 24 hours post seeding, HOBs were treated with a Wnt agonist, CHIR (5 nM) (Selleckchem), ABC (0.5 μg/mL), or DMSO (vehicle control) for 24 hours. Following fixation, cells were stained to assess total β-catenin (1:100, Sigma-Aldrich), hypo-phosphorylated (active) β-catenin (1:100, USBiological), anti-rabbit α-tubulin (1:100, Cell Signaling), or anti-mouse α-tubulin (1:100, Boster Bio). Detailed methods are presented in the supplement.

### TOPFlash: β-catenin reporter plasmid

Early passage HOBs were transduced with TOPFlash lentivirus and selected with puromycin (2 μg/mL, Sigma-Aldrich). Luciferase activity was confirmed using the dual luciferase assay reporter system (Promega Madison, WI, USA). To verify Wnt/β-catenin signaling pathway activation in HOBs, cells were treated with Wnt agonists, CHIR (5 nM), and Wnt3a (2 ng/mL).

### Mass spectrometry

Antiretroviral concentrations in bone tissue (bone with bone marrow intact, femur) and bone matrix (bone without bone marrow, right and left tibiae) were measured with mass spectroscopy. Dolutegravir, TFV-diphosphate (TFV-DP; TDF intracellular anabolite), carbovir triphosphate (CBV-TP, ABC intracellular anabolite), 3TC triphosphate (3TC-TP, 3TC intracellular anabolite) were evaluated using validated LC/MS/MS methods.^(^^31–33^^)^

### Bone mineral density

Whole body, right femur, and lumbar spine (L4-6) BMD (g/cm^2^) were measured using dual-energy X-ray absorptiometry (DXA, Kubtek Stratford CT). Mice were anesthetized using isoflurane and placed in the DXA machine in the prone position for scanning.

### Micro-computed tomography

Right femurs were micro-computed tomography (μCT) scanned while submerged in 70% ethanol. Scanning parameters were 55 kVp and 145 μA, with 500 ms integration time and a 6 μm isotropic voxel size (μCT50, Wangen-Brüttisellen, Switzerland). The middle 100 slices of the femoral diaphysis were evaluated to assess cortical area (Ct.Ar), total area (Tt.Ar), medullary area (Ma.Ar), cortical thickness (Ct.Th), and cortical porosity (Ct.Po). A region between the distal 30% of the total femoral length to the proximal growth plate was used to assess bone volume per total volume (BV/TV), trabecular number (Tb.N), trabecular thickness (Tb.Th), and trabecular spacing (Tb.Sp).

### 3-point bend mechanical loading test

Femurs were loaded to failure in the anterior–posterior direction using a lower support span length of 10 mm, a loading rate of 0.1 mm/s, and a data acquisition rate of 100 Hz (MTS CriterionTM). A preload of ~0.25 N was applied to prevent shifting during testing. Load–displacement curves were used to determine the peak load and bending stiffness.

### Circulating bone turnover markers and proinflammatory cytokines

Circulating markers were measured using enzyme-linked immunosorbent assay (ELISA). Circulating total procollagen type 1 N-terminal propeptide (P1NP, Immunodiagnostics Systems, Inc., Fountain Hills, AZ, USA), C-terminal telopeptide of type I collagen (CTX, Immunodiagnostics Systems), sclerostin (R&D Systems, Minneapolis, MN, USA), interleukin-6 (IL-6, R&D Systems) and tumor necrosis factor-alpha (TNFα R&D Systems).

### Statistical analysis

Statistical analyses were performed using Prism software (GraphPad Prism, San Diego, CA, USA). Data were first confirmed to be normally distributed. Group comparisons were made using one-way analysis of variance (ANOVA) tests. For *in vitro* experiments, one-way ANOVAs were used to compare the effects of ARV concentrations and for *in vivo* experiments, one-way ANOVAs were used to compare the effects of various treatments, *post hoc* comparisons were performed using two-way 2-sample T-tests when appropriate.

## Results

### ARVs impair osteoblast function, without affecting cell viability

TDF, ABC, DTG, and 3TC individually, had minimal effects on cell viability (Supplementary Figure 1). Osteoblast function was assessed using Alizarin red staining (ARS) and ALP activity staining (Supplementary Figure 2). Both TDF concentrations reduced ARS and 0.64 μg/mL reduced ALP activity staining compared to DMSO. Abacavir at 4 μg/mL decreased ARS but did not affect ALP activity. DTG at 0.48 μg/mL decreased both ARS and ALP staining. 3TC at 0.15 μg/mL increased ARS but not ALP activity compared to DMSO. Reduced ARS and ALP activity was observed with the high-dose ABC/DTG/3TC combination.

### ARVs alter osteoblast gene expression

**Figure 1:**
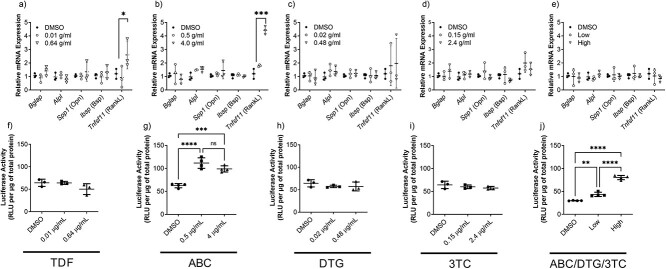
Expression of osteoblastic genes: *Bglap, Alpl, Spp1 (osteopontin or Opn), Ibsp (bone sialoprotein or Bsp2)* and *Tnfsf11* (*RankL)* mRNA expression in HOBs treated with (a) TDF, (b) ABC, (c) DTG, (d) 3TC, and (e) ABC/DTG/3TC combination. Wnt/β-catenin signaling pathway activation as measured by luciferase activity in TOPFlash transfected HOBs treated with (f) TDF, (g) ABC, (h) DTG, (i) 3TC, and (j) ABC/DTG/3TC.Data are presented as mean ± standard deviation of 3 independent experiments (biological replicates) each performed in triplicate (technical replicates). Data were analyzed with a one-way analysis of variance (ANOVA) followed by post-hoc comparisons, when appropriate. ^*^p ≤ 0.05, ^**^p ≤ 0.01, ^***^p ≤ 0.001.

The impact of treatment on the gene expression of osteoblasts were ARV and dose-dependent (Figure 1). TDF at 0.64 μg/mL, but not at 0.1 μg/mL, increased *RANKL* compared to controls. Similarly, ABC increased *RANKL* expression, but only at the highest concentration tested. No other genes were affected by TDF or ABC and none of the genes were affected by DTG, 3TC, or ABC/DTG/3TC.

### Abacavir activates Wnt/β-catenin transcriptional activity

To evaluate the effects of ARV treatment on Wnt/β-catenin transcriptional activity, HOBs were transduced with a TOPFlash containing lentivirus. As a positive control, TOPFlash transduced fully differentiated HOBs were treated with Wnt-agonists, CHIR, or Wnt3a, which increased luciferase activity approximately 120-and 6-fold compared to DMSO (Supplementary Figure 3). TDF, DTG, and 3TC did not affect Wnt/β-catenin transcriptional activity when compared to controls (Figure 1). Abacavir increased Wnt/β-catenin signaling by 79 and 58%, for 0.5 and 4.0 μg/mL concentrations, respectively. ABC/DTG/3TC combination, induced Wnt/β-catenin signaling by 46 and 168%.

**Figure 2:**
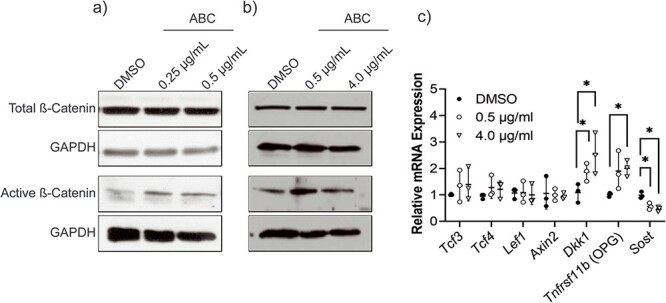
Total and active β-catenin protein expression and the mRNA expression of Wnt/β-catenin related genes in HOBs treated with ABC. (a) Representative total and hypo-phosphorylated (active) β-catenin western blot with 0.25 and 0.5 μg/ml of ABC or DMSO. (b) Representative total and active β-catenin western blot with 0.5 and 4.0 μg/ml of ABC or DMSO. (c) mRNA expression of *Tcf3, Tcf4, Lef1, Axin2, Dkk1, Tnfrsf11b (osteoprotegrin, Opg), Sost*. Relative mRNA expression is presented as mean ± standard deviation of 3 independent experiments (biological replicates) each performed in triplicate (technical replicates). Data were analyzed with a one-way analysis of variance (ANOVA) followed by post-hoc comparisons, when appropriate. ^*^p ≤ 0.05, ^**^p ≤ 0.01, ^***^p ≤ 0.001.

Abacavir did not affect total β-catenin protein levels when compared to DMSO but did increase the levels of hypo-phosphorylated β-catenin (Figure 2). Abacavir increased *Opg (Tnfrsf11b)* and *Dkk1* expression and reduced *Sost* expression. While ABC did not affect total β-catenin (Supplementary Figure 4), hypo-phosphorylated β-catenin nuclear staining intensity increased in ABC-treated HOBs compared DMSO (Supplementary Figure 5).

### ARV accumulation in bone tissue

**Figure 3:**
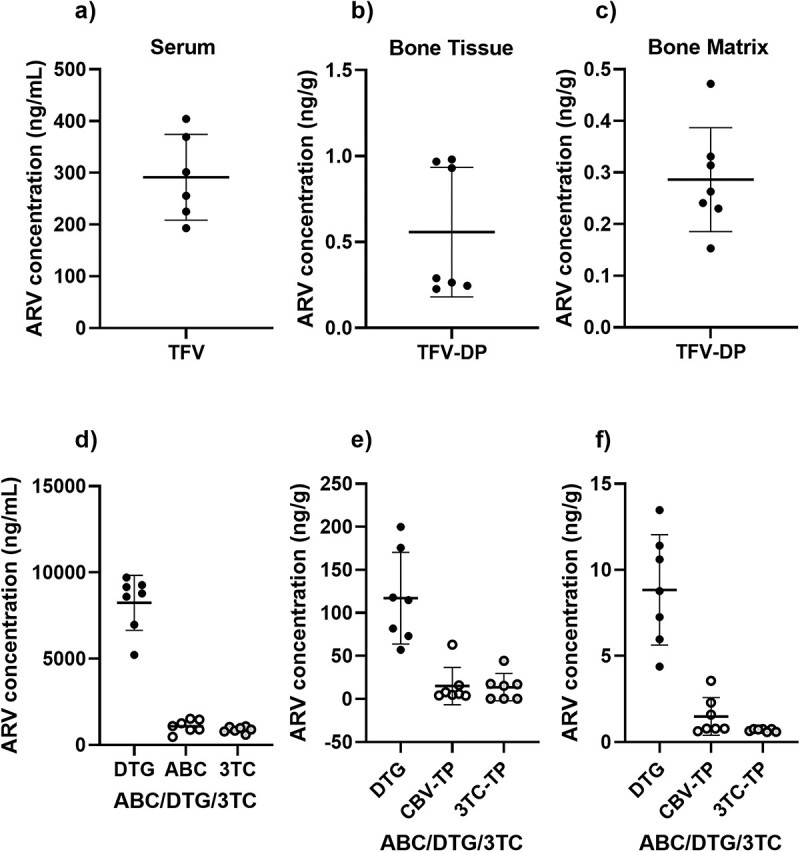
Antiretroviral (ARV) concentrations measured in the serum (a, d), femurs containing bone marrow (bone tissue, b, e), or tibiae depleted of bone marrow (bone matrix, c, f). Data are presented as the mean ± standard deviation for each group, with a total sample size of TDF, *n* = 7 and ABC/DTG/3TC, *n* = 7.

Adult, 12-week-old, male C57BL/6 J mice received orally administered TDF or ABC/DTG/3TC combination. At 2-weeks (see Supplemental materials), and 4 weeks (Figure 3) post-initiation, all ARVs were detected in circulation. ARV concentrations in bone tissue were evaluated using intact femurs containing bone marrow. The concentrations of TVP-DP, CBV-TP, DTG, and 3TC-TP in the bone tissue were 0.6, 117, 15.1, and 13.7 ng/g or roughly 0.18%, 1.4%, 1.4%, and 1.5% of the circulating doses, respectively. However, 3 of 7 bone tissue samples measured below the lower limit of detection for 3TC-TP. In the bone matrix, the concentrations of TVP-DP, CBV-TP, and DTG were 0.3, 8.8, and 1.5 ng/g or roughly 0.09%, 0.14%, and 0.11% of the circulating levels. Although, 4 of 7 bone matrix samples measured below the lower limit of detection for CBV-TP. All the bone matrix samples measured below the lower limit of detection for 3TC-TP. The tissue density of adult bone is approximately 2.0 g/cm^3^ or 2.0 g/mL.^(^^34^^)^ Therefore, the tissue concentrations of each ARV tested are converted to g/mL and presented in Supplementary Table 1.

### TDF but not ABC/DTG/3TC decreased lumbar spine BMD in vivo

The *in vivo* effects of ABC/DTG/3TC were evaluated in uninfected male C57BL/6J mice and compared to TDF (Supplementary Table 2). Neither TDF nor ABC/DTG/3TC affected body weight. Similarly, neither treatment affected total body BMD. Tenofovir disoproxil fumarate reduced lumbar spine BMD compared to both the control and ABC/DTG/3TC treated mice. Neither TDF nor ABC/DTG/3TC combination treatment affected femoral BMD.

### ABC/DTG/3TC reduced femoral microarchitecture while TDF decreased vertebral connectivity compared to controls

At the femur, ABC/DTG/3TC treated mice had decreased cortical area compared to controls. Both TDF and ABC/DTG/3TC treated mice had decreased cortical thickness compared to controls. Total area, medullary area and cortical porosity were unaffected by treatment (Supplementary Table 2). In the distal femur, the trabecular thickness was reduced in ABC/DTG/3TC treated mice compared to controls, but no other femoral parameters were different. In the lumbar spine, the connectivity density was reduced in the TDF-treated mice compared to controls. The ABC/DTG/3TC treated mice had reduced trabecular spacing and thickness but increased trabecular number and connectivity density when compared to TDF mice, but no differences when compared to controls. Antiretroviral had no effect on the femoral mechanical properties (Supplementary Table 2).

### ARVs reduced circulating bone turnover markers

Circulating P1NP, CTX, and sclerostin were evaluated to assess ARV effects on bone remodeling (Supplementary Table 2). P1NP or CTX levels were not different between groups; however, sclerostin was decreased in TDF-treated mice compared to controls. While pro-inflammatory cytokine levels were assessed, both IL-6 and TNFα were below the detection limit for nearly all samples (see Supplemental materials for more information).

### Abacavir activates the Wnt/β-catenin signaling pathway and increases bone formation in vivo

Due to the positive effects of ABC on Wnt/β-catenin signaling in vitro, we performed a third *in vivo* study to evaluate the effects of ABC monotherapy in mice. Abacavir did not affect body weight, or total body, lumbar spine, or right femoral BMD (Supplementary Table 3). Trabecular microarchitecture, cortical geometry, and femoral mechanical properties were also not affected by ABC.

**Figure 4:**
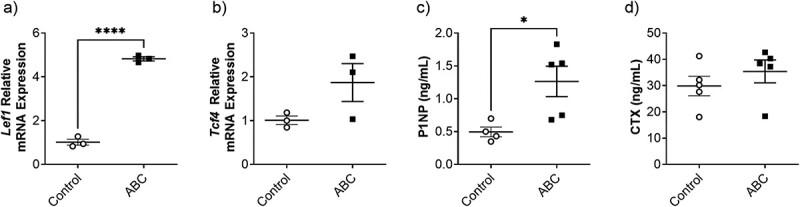
Skeletal gene expression of (a) *Lef1* and (b) *Tcf4* measured in the osteocyte-enriched samples from the tibial diaphysis and circulating (c) P1NP and (d) CTX of control and ABC treated male C57BL6/J mice. Data are presented as the mean ± standard deviation for each group with a total sample size of control *n* = 3, and ABC *n* = 3-4. Data were analyzed with unpaired t-tests. ^*^p ≤ 0.05. ^*^p ≤ 0.05, ^**^p ≤ 0.01, ^***^p ≤ 0.001, ^****^p ≤ 0.0001.

Despite the limited impact on bone structure, ABC activated the skeletal expression of Wnt/β-catenin genes (Figure 4). Specifically, osteocyte-enriched cortical extracts from ABC-treated mice had significantly increased *Lef1* and a trend towards higher *Tcf4* expression compared to controls. P1NP was significantly increased in ABC-treated mice, while CTX was not affected by ABC treatment.

## Discussion

Our study examined the HIV-independent effects of DTG and ABC, two ARVs with relatively limited data on their skeletal effects. We combined *in vitro* and *in vivo* studies to determine the effects of TDF and ABC independently and the combination of ABC/DTG/3TC on bone cells, mass, quality, and strength. We found that ABC/DTG/3TC has negative effects on the skeleton in the absence of HIV, although the magnitude is small. Therefore, suggesting that increased bone mass in PLWH switching from TDF to DTG^(^^12^^)^ or ABC^(^^10^^,^^11^^)^ is more a reflection of TDF cessation. However, we also note that ABC activates Wnt/β-catenin signaling in bone cells *in vivo* and *in vitro*, with evidence for activated bone formation following ABC treatment. Our data did not find that ABC treatment lead to increased bone mass, therefore, whether ABC-induced Wnt/β-catenin signaling activation has net positive or negative effects on bone requires further study.

We directly compared the *in vivo* effects of TDF and ABC/DTG/3TC independent of HIV and confirmed that TDF reduced lumbar spine BMD, while ABC/DTG/3TC did not affect BMD. Tenofovir disoproxil fumarate-induced loss of BMD, independent of HIV infection, is consistent with data from cART prophylaxis studies.^(^^35^^)^ Interestingly, we did not find elevated levels of the bone resorption marker, CTX, coincidence with the loss of BMD. Clinically, use of TDF based cART for prophylaxis (ie independent of HIV), is generally associated with elevated CTX levels.^(^^36^^,^^37^^)^ However, data from preclinical models have been more mixed. For example, both Conradie et al^(^^38^^)^ and Matuszewsaka et al^(^^23^^)^ noted bone loss with TDF treatment in rats, but neither detected elevated bone resorption marker levels. The reason for this discrepancy is unknown but has been noted in the clinic as well, with direct tissue assessments of bone biopsies from PLWH initiating TDF-based cART showing increased osteoclast surfaces with no corresponding change in CTX levels.^(^^39^^)^ Unfortunately, we were not able to assess resorption at the tissue level in the current study. Future studies are necessary to evaluate the timing of TDF-induced osteoclast activity at both the tissue and systemic levels.

While we assessed levels of proinflammatory cytokines previously linked to cART-induced bone loss in PLWH,^(^^6^^)^ the levels were largely undetectable, suggesting that inflammation may be less important to HIV-independent cART-induced bone loss. However, despite reduced BMD, minimal changes were noted in trabecular microarchitecture, with only connectivity density in the lumbar spine significantly reduced in TDF-treated mice. It is worth noting that high resolution peripheral quantitative computed tomography (HRpQCT) studies have also reported no microarchitectural changes in PLWH with BMD loss.^(^^40^^)^ In further support of our results, Foreman et al^(^^41^^)^ reported that changes in bone microarchitecture were only seen clinically when TDF was used in combination with a protease inhibitor. To our knowledge, no clinical studies have yet evaluated the effects of ABC/DTG/3TC on bone microarchitecture. Trabecular bone score, as assessed by DXA, is reported to decrease in response to ABC/DTG/3TC treatment.^(^^16^^)^ However, it is difficult to determine how this relates to the findings in the current study, which noted primarily cortical bone changes in the mid-diaphysis in response to ABC/DTG/3TC.

Although the combination of ABC/DTC/3TC was not bone stimulatory, ABC monotherapy increased Wnt/β-catenin signaling in osteoblasts *in vitro* and osteocyte-enriched bone tissue *in vivo*. While previous studies in Wister rats have noted increased trabecular bone mass with ABC treatment,^(^^23^^)^ in the current study, we found no changes in BMD or microarchitecture in ABC-treated mice. The discrepancies could be explained by differences in treatment duration, 6-weeks vs 16 weeks in Matuszewska et al.^(^^23^^)^ ABC increased P1NP, a marker of bone formation, which supports the possibility of bone mass accumulation with longer treatment, however, as described below, the activation of Wnt/β-catenin signaling does not always lead to increased bone mass. Interestingly, we did not detect any changes in β-catenin in TDF treated cells, which was reported by Conesa-Beundia et al.^(^^42^^)^ The reason(s) are unclear but may be explained by the differences in concentrations tested, the source of the osteoblasts (human vs murine), or the source of TDF – tenofovir in Conesa-Beundia and TDF in our study.

Despite increased Wnt/β-catenin signaling transcriptional activity and increased active β-catenin protein expression in osteoblasts treated with ABC, there was reduced matrix calcification *in vitro*. Although, these would seem contradictory, there is evidence that chronic β-catenin activation can have deleterious effects on bone cells. For instance, constitutive activation of β-catenin promotes osteoblast proliferation but inhibits osteoblast differentiation and decreases mineral accumulation *in vitro*.^(^^43–48^^)^ Mice harboring mutations that lead to constitutive β-catenin activation have impaired bone growth, lower bone mass, decreased matrix mineralization and a disorganized collagen matrix.^(^^43^^)^ Therefore, our data suggest that ABC-induced activation of Wnt/β-catenin likely inhibits osteoblast differentiation, which may explain the lack of increased ARS *in vitro* or bone mass *in vivo*. Further work is needed to determine the long-term effects of ABC treatment alone or in combination cART formulations on Wnt signaling, bone remodeling, matrix mineralization, and bone strength.

Bone is difficult to target pharmaceutically^(^^49^^)^ and yet highly responsive to ARVs.^(^^50^^)^ Our study is the first study to measure the skeletal distribution of ARVs in bone matrix, which includes primarily osteocytes, and bone tissue, which includes bone marrow, a source for immune cells targeted by HIV, including CD4+ T-cells.^(^^51^^)^ We show that DTG accumulates at the highest concentration, followed by TDF, which both have higher levels in bone tissue than in bone matrix. Although it is possible that longer-term treatment may increase the accumulation of ARVs, particularly in the extracellular matrix of the bone, we opted for 6-weeks, which is similar to published mouse studies of TDF exposure as a starting point.^(^^42^^,^^52^^)^ The tissue concentrations measured are considerably lower than those commonly used *in vitro*, including within the current study. For our *in vitro* experiments, we chose concentrations that matched circulating levels, while others have used IC90 levels needed to inhibit viral replication^(^^53^^)^ or sublethal *in vitro* concentrations.^(^^54–56^^)^ While our data largely conforms with published *in vitro* data, such as decreased ARS due to TDF^(^^54^^)^ and DTG,^(^^53^^)^ the *in vivo* distribution data suggest that future work is needed to confirm these results at lower concentrations.

Our study strengths include employing a cART regimen (DTG/ABC/3TC) currently endorsed as first-line treatment in the United States.^(^^57^^)^ The design involved measuring ARV concentrations in bone tissue, enhancing the blueprint for future *in vivo* studies. Antiretrovirals were administered orally and in combination, replicating clinical cART use. Weaknesses include the use of 12-week-old mice, which although past the period of longitudinal bone growth, may still be undergoing some degree of bone mass expansion and relatively short treatment duration, potentially limiting observable impacts. However, we were primarily interested in the immediate effects of initiation, as BMD changes are largest in PLWH initiating cART.^(^^50^^)^

Combined antiretroviral therapy effects were evaluated in the absence of HIV, which may synergize with cART to increase BMD loss.^(^^58^^)^ The lack of HIV may also help explain the relatively small effect sizes detected in the bone metrics, as BMD loss with TDF use as prophylaxis have been reported to be small (~1% loss of BMD in uninfected TDF users^(^^59^^)^) and therefore future work with humanized mouse models will be important to determine the potential additive effects of HIV. Lastly, the effects of ARVs were only evaluated in bone forming osteoblasts.

## Conclusions

In summary, a negative effect of ABC/DTG/3TC was detected in both primary osteoblasts and in bone tissue from uninfected C57BL/6J mice. We also noted that ABC treatment activates Wnt/β-catenin both *in vitro* and *in vivo* but does not appear to increase the formation of mineralized bone matrix in either model system. Overall, our findings show the reported bone mass increase following TDF switch is not due to bone stimulatory effects of newer formulation ARVs but more likely due to cessation of bone toxic TDF.

## Author contributions

Arnold Z. Olali (Conceptualization, Formal analysis, Writing—original draft, Writing—review editing), Jennille Wallace (Conceptualization, Formal analysis, Writing—review editing), Hemil Gonzalez (Conceptualization, Formal analysis, Writing—review editing), Kelsey A. Carpenter (Formal analysis, Writing—review editing), Niyati Patel (Formal analysis, Writing—review editing), Lee C. Winchester (Formal analysis, Writing—review editing), Anthony T. Podany (Conceptualization, Project administration, Supervision, Writing—review editing), Ishwarya Venkatesh (Formal analysis, Writing—review editing), Srinivasa D. Narasipura (Conceptualization, Formal analysis, Writing—review editing), Lena Al-Harthi (Conceptualization, Project administration, Supervision, Writing—review editing), and Ryan D. Ross (Conceptualization, Project administration, Supervision, Resources, Funding acquisition, Writing—review editing)

## Funding

Research reported in this publication was supported by the National Institute of Arthritis and Musculoskeletal and Skin Diseases of the National Institutes of Health under award numbers: R21AR079309 (R.D.R.) and R01AR081151 (R.D.R.). The content is solely the responsibility of the authors and does not necessarily represent the official views of the National Institutes of Health.

## Conflicts of interest

The authors declare no competing or financial interests.

## Data availability

The data underlying this article are available in the article and in its online supplementary material.

## Supplementary Material

Olali_et_al_ABC_and_Wnt_Supplemental_ziae037
