# Supplementary material for: The anti-HIV drug abacavir stimulates β-catenin activity in osteoblast lineage cells
Source: JBMR Plus. 2024 Mar 19;8(5):ziae037. doi: 10.1093/jbmrpl/ziae037 (PMC11001392; doi:10.1093/jbmrpl/ziae037)
Supplement: Olali_et_al_ABC_and_Wnt_Supplemental_ziae037 [file olali_et_al_abc_and_wnt_supplemental_ziae037.docx]

**The Anti-HIV Drug Abacavir stimulates β-catenin activity in osteoblast lineage cells**

Arnold Z. Olali^1,2^, Jennillee Wallace^2^, Hemil Gonzalez^2,3^, Kelsey A Carpenter^1^, Niyati Patel^1^, [Lee C Winchester](about:blank)^4^, [Anthony T Podany](about:blank)^4^, Ishwarya Venkatesh^5^, Srinivas D. Narasipura^2^, Lena Al-Harthi^2^, Ryan D Ross^1,2,6^

1. Department of Anatomy & Cell Biology, Rush University Medical Center, Chicago, IL, USA.
2. Department of Microbial Pathogens and Immunity, Rush University Medical Center, Chicago, Illinois, USA.
3. Department of Internal Medicine, Division of Infectious Diseases, Rush University Medical Center, Chicago IL, USA
4. UNMC Center for Drug Discovery, University of Nebraska Medical Center, Omaha, NE, USA
5. Department of Internal Medicine, Drug Discovery Center, Rush University Medical Center, Chicago, IL, USA
6. Department of Orthopedic Surgery, Rush University Medical Center, Chicago, IL, USA

**Supplemental Materials.**

**Methods**

**Osteoblasts culture and differentiation**

HOBs were cultured in alpha-modified essential medium (αMEM) with Earl’s salt, ribonucleosides, deoxyribonucleosides & L-glutamine (Conning, [Hartford, CT](about:blank)), supplemented with 10% fetal bovine serum (Atlanta Biosciences), 100 U/mL penicillin, 100 μg/mL streptomycin (ThermoFisher), hereafter referred to as complete media. New media was replaced every 3 days. Differentiation was induced using osteogenic media, which consisted of complete media supplemented with 50 µg/mL L-ascorbic acid (Millipore Sigma), 10 mM β-glycerophosphate (Millipore Sigma), and 100 nM dexamethasone (Millipore Sigma).

**C57BL/6J mice**

Animals in the first experiment were used to measure bone tissue ARV concentrations. Mice received tail vein blood draws 2 weeks after treatment initiation to confirm ARVs were detectable in circulation. At euthanasia, right femurs were isolated, cleaned of all soft tissues, snap-frozen intact in liquid nitrogen, without removing bone marrow, and stored at -80°C until use. The left and right tibiae were cleaned of all soft tissue and the proximal and distal epiphyses were removed. The resulting diaphysis was centrifuged at 5,000 rpm for 10 min at 4°C to remove the medullary content and flushed with PBS. The resulting bone matrix samples were snap-frozen in liquid nitrogen and stored at -80°C until use.

In experiments two and three, right femurs and lumbar vertebrae were collected and fixed in 10% neutral buffered formalin. Left femurs were wrapped in phosphate buffer saline (PBS) and stored at -20^o^ C. Blood was collected via cardiac puncture and allowed to clot at room temperature for 30 minutes before being centrifuged at 3,400 rpm for 15 min at 4°C to separate serum. The right and left tibiae were cleaned of all soft tissue and the proximal and distal epiphyses were removed, centrifuged at 5,000 rpm for 10 min at 4°C to remove the medullary content, flushed with PBS, and submerged in RNAlater (Ambion).

**Cell viability**

On day 14, 10μL of the MTT reagent (5 mg/mL, EMDmillipore, Bedford, MA, USA) was added to each well, and plates were incubated at 37°C for 4 hours. The assay was read at 570 nm using a Synergy HT microplate reader using Gen5 software (BioTek, Winooski, VT). The experiment was repeated three separate times, with no differences between the results of each independent experiment.

**Detection and quantification of alizarin red staining (ARS)**

On day 14, HOBs were washed with PBS and fixed with 4% (v/v) formaldehyde (Sigma-Aldrich) for 15 min at room temperature. Following fixation, HOBs were washed with dH2O. 40 mM Alizarin red staining (ARS) solution at pH 4.2 (Sigma-Aldrich) was added to each well. Plates were incubated at room temperature for 10 min. The ARS solution was aspirated, and wells were washed 3 times with dH2O while shaking. Plates were air dried and stored at −20°C for quantification of bound alizarin red. Bound alizarin red was extracted and quantified in triplicate at 405 nm in a 96-well format using Synergy HT microplate reader using Gen5 software (BioTek, Winooski, VT).

**Detection and quantification of alkaline phosphatase (ALP) activity staining**

On day 14, HOBs were washed with PBS and exposed to 1 mL of 1-step NBT/BCIP Substrate Solution (ThermoFisher Scientific) then incubated at room temperature for 10 min. The 1-step NBT/BCIP was aspirated, and HOBs were washed 3 times with dH2O and then air dried at room temperature. ALP staining intensity was quantified using Image J version 2.1 (NIH, Bethesda, MD) and normalized to DMSO treated HOBs.

**Quantitative real-time PCR**

RNA was isolated on day 14 using Trizol (Ambion) followed by storage at -80^o^ C. Tibia from ARV treated mice crushed with a mortar and pestle, submerged in Trizol, and homogenized with a Polytron PT 10–35 Homogenizer (Brinkmann). Total mRNA was extracted according to the Trizol manufacturer’s protocol, incubated with Dnase I (Sigma, St. Louis, MO). cDNA was synthesized using Qscript supermix (Quanta Biosciences, Beverly, MA). Real-time PCR was performed using SSO fast SYBR green supermix (Biorad, Hercules, CA) in a 7500 real-time PCR system (Applied Biosystems, Waltham, MA).

**Western Blot**

Twenty to thirty micrograms of total cell lysate was separated using 10% SDS-PAGE, transferred onto a nitrocellulose membrane, blocked with superblock (ThermoFisher) containing 0.1% Tween 20 (T20) for 1 h. The nitrocellulose membrane was then incubated with primary antibody for total β-catenin (1:10,000, Sigma-Aldrich), hypo-phosphorylated (active) β-catenin (1:1000, USBiological), Glyceraldehyde 3-phosphate dehydrogenase (GAPDH, Sigma-Aldrich) overnight at 4°C in superblock-0.1% Tween-20 (T20). Membranes were then washed for 10 min 3 times with TBS-T20 then incubated with a secondary antibody conjugated to horseradish peroxidase (1:5,000) (HRP, Cell Signaling) in superblock-0.1% T20 for 1 hour at RT. Membranes were again washed for 10 min 3 times with TBS-T20 and developed with super signal west femto maximum sensitivity substrate (ThermoFisher) according to the manufacturer’s instructions.

**Immunofluorescence staining**

HOBs were then fixed with 4% paraformaldehyde (PFA, pH 7.4) for 10 min at 37°C. PFA was aspirated and cells were washed 3 times with 1x PBS before being permeabilized in 0.1% Triton X-100 in 1X PBS at room temperature for 15 mins. After permeabilization, HOBs were washed with 1x PBS and blocked using 2% bovine serum albumin (BSA) in 1x PBS at room temperature for 1 hour. HOBs were then incubated in primary antibodies at 4°C for 24 hrs. Primary antibodies used included anti-rabbit total β-catenin (1:100, Sigma-Aldrich), anti-mouse hypo-phosphorylated (active) β-catenin (1:100, USBiological), anti-rabbit α-tubulin (1:100, Cell Signaling), or anti-mouse α-tubulin (1:100, Boster Bio) in 0.1% BSA. HOBs were then washed 3 times for 10 min each with Tris-buffered saline with 0.1% Tween® 20 Detergent (TBST). Secondary antibodies included anti-rabbit IgG (H+L Alexa Flour®594, 1:100, ThermoScientific), anti-rabbit IgG (H+L Alexa Fluor® 488 1:100, ThermoScientific), anti-mouse IgG H&L (Alexa Fluor® 488 1:100, Abcam), and anti-mouse IgG (H&L Alexa Fluor® 594, 1:100, Abcam). After incubation with the secondary antibodies, the HOBs were then washed with 1X TBST. The coverslips were mounted on glass slides using Mounting Medium with DAPI – Aqueous, Fluor shield (Abcam).

Immunofluorescence images were acquired using a Nikon Eclipse TI2-E inverted microscope and subsequently processed using NIS-elements software (Nikon Corporation, Tokyo, Japan). CellProfiler version 3.1.9 was utilized for quantifying fluorescent intensity. In brief, a customized pipeline comprising multiple modules was constructed for analysis. Initially, all image sets were converted to grayscale to align with the analysis pipeline. Nuclei were identified as the starting points to pinpoint the cells using the “Identify Primary Objects” module. Subsequently, using the “Secondary and Tertiary Object” modules, nuclei were extended to define cell borders and cytoplasm. The fluorescence intensity of each object identified in the preceding modules was measured via the “Measure Object Intensity” module, which extracted pixel intensity and reported it as mean intensity units.

**TOPFlash: β-catenin reporter plasmid**

Second generation lentivirus (LV) particles containing TOPFlash RNA was generated of high-quality endotoxin free plasmid, the 7TFP (transfer plasmid, Addgene), pCMV-VSV-G (Addgene), and pMD2 (Addgene) into 293T cells using calcium phosphate transfection reagent (Invitrogen) according to the manufacturer’s protocol. To measure luciferase activity, HOBs were harvested and lysed with passive lysis buffer by incubating at 37°C for 10 min. The cell lysates were spun at 5,000 rpm for 4 min to remove debris. Luciferase activity was measured in 20 μL of total cell lysate using the dual luciferase assay reporter system (Promega Madison, WI). Total protein content was measured using BCA and relative light units were normalized to μg/mL of protein. DMSO treatment was used as a vehicle control.

**Mass spectrometry**

In preparation for mass spectrometry, bone tissues were thawed, weighed, and homogenized in 0.5 mL of 70% methanol with a Precellys Evolution Cryolys homogenizer (Bertin Technologies, USA) in a temperature-controlled chamber (-20°C) according to the manufacturer’s protocol. Tissue homogenates were centrifuged at 10,000 rpm for 20 min and supernatants containing drugs were collected into fresh tubes and stored at -80°C until used for drug quantification.

**Results.**

**ARV accumulation in bone tissue**

At 2 weeks post initiation, the active tenofovir metabolite of TDF, tenofovir (TFV) was detected in the serum at a mean (SD) concentration of 213 (±37) ng/mL. In mice receiving ABC/DTG/3TC, CBV, the active ABC metabolite, was 553 (±486) ng/mL, DTG was 7,004 (±1,345) ng/mL, and 3TC was 263 (±106) ng/mL.

**Proinflammatory Cytokines**

In control mice, each animal was below the levels of detection for both IL-6 and TNFα (1.8 pg/mL and 7.21 pg/mL, respectively). Only one of the 7 available TDF serum samples had detectible levels in either assay (3.13 and 1.05 pg/mL for IL-6 and TNFα, respectively). In the ABC/DTG/3TC animals, none had detectible levels of IL-6, while only one had a measurement above the detection limit, which was 4.89 pg/mL.

**Supplemental Table 1:** Human primer sequences used for human osteoblast (HOB) experiments.

| β-Actin Forward | CATGTACGTTGCTATCCAGGC |
| --- | --- |
| β-Actin Reverse | CTCCTTAATGTCACGCACGAT |
| BGLAP Forward | CACTCCTCGCCCTATTGGC |
| BGLAP Reverse | CCCTCCTGCTTGGACACAAAG |
| ALPL Forward | ACCACCACGAGAGTGAACCA |
| ALPL Reverse | CGTTGTCTGAGTACCAGTCCC |
| BSP2 Forward | CACTGGAGCCAATGCAGAAGA |
| BSP2 Reverse | TGGTGGGGTTGTAGGTTCAAA |
| TNFSF11 Forward | CAACATATCGTTGGATCACAGCA |
| TNFSF11 Reverse | GACAGACTCACTTTATGGGAACC |
| TCF-3 Forward | CCGACTCCTACAGTGGGCTA |
| TCF-3 Reverse | CGCTGACGTGTTCTCCTCG |
| TCF-4 Forward | CAAGCACTGCCGACTACAATA |
| TCF-4 Reverse | CCAGGCTGATTCATCCCACTG |
| LEF-1 Forward | AGAACACCCCGATGACGGA |
| LEF-1 Reverse | GGCATCATTATGTACCCGGAAT |
| Axin2 Forward | TACACTCCTTATTGGGCGATCA |
| Axin2 Reverse | TTGGCTACTCGTAAAGTTTTGGT |
| Dkk1 Forward | CCTTGAACTCGGTTCTCAATTCC |
| Dkk1 Reverse | CAATGGTCTGGTACTTATTCCCG |
| OPG Forward | GCGCTCGTGTTTCTGGACA |
| OPG Reverse | AGTATAGACACTCGTCACTGGTG |
| SOST Forward | ACACAGCCTTCCGTGTAGTG |
| SOST Reverse | GGTTCATGGTCTTGTTGTTCTCC |

**Supplemental Table 2:** Murine primer sequences used for mouse experiments.

| β-Actin Forward | CATTGCTGACAGGATGCAGAAGG |
| --- | --- |
| β-Actin Reverse | TGCTGGAAGGTGGACAGTGAGG |
| LEF-1 Forward | ACTGTCAGGCGACACTTCCATG |
| LEF-1 Reverse | GTGCTCCTGTTTGACCTGAGGT |
| TCF-7 Forward | CGCTGACAGTCAACGCATCTATG |
| TCF-7 Reverse | GGAGGATTCCTGCTTGACTGTC |


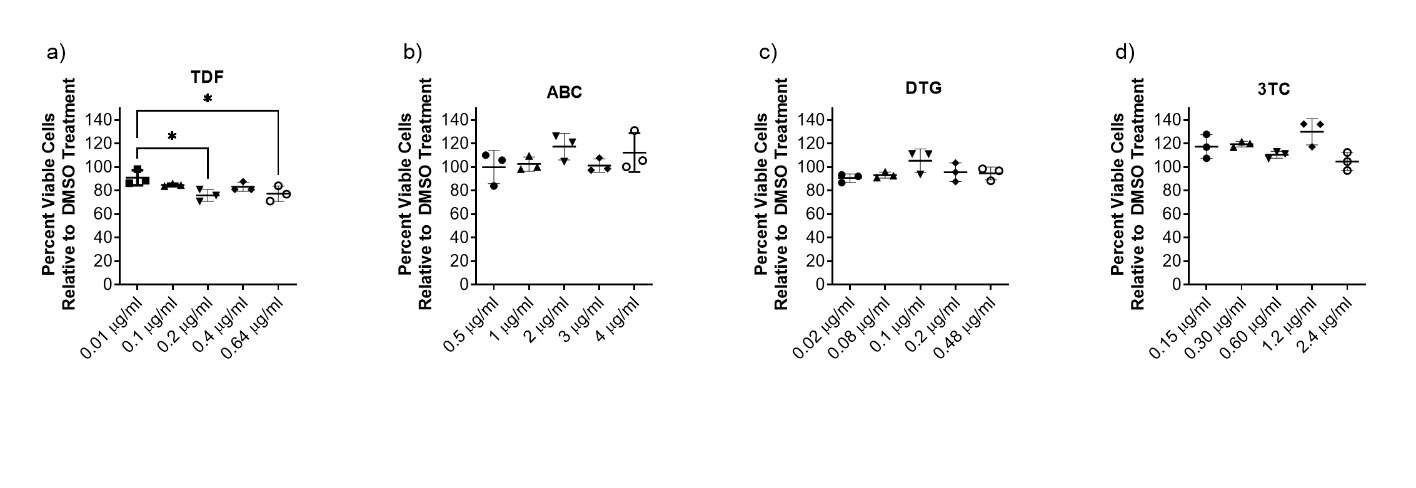


**Supplemental Figure 1**: Cell viability following chronic treatment with **(a)** TDF, **(b)** ABC, **(c)** DTG, and **(d)** 3TC. Cell viability was determined using the MTT assay. Data are presented as the mean ± standard deviation of 3 independent experiments (biological replicates or wells) and represent one of three independent replicates of the MTT assay. Data were analyzed with a one-way analysis of variance (ANOVA) followed by post-hoc comparisons, when appropriate. * p ≤ 0.05 ** p ≤ 0.01, *** p ≤ 0.001.

**
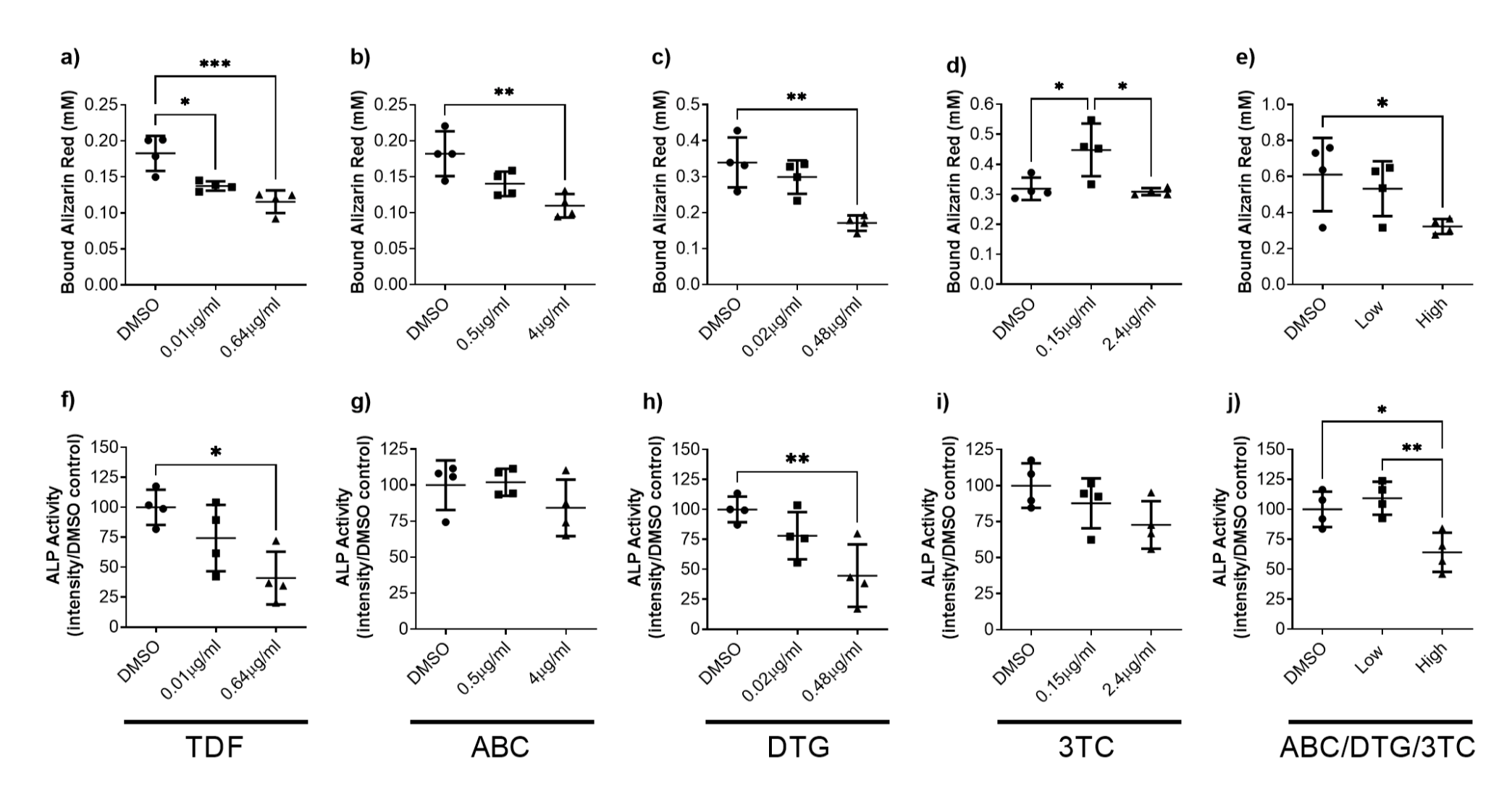
**

**Supplemental Figure 2**: Alizarin red staining and alkaline phosphatase (ALP) activity quantification following chronic exposure to (a, f) TDF, (b, g) ABC, (c, h) DTG, (d, i) 3TC and (e, j) ABC/DTG/3TC. Low dose ABC/DTG/3TC is composed of 0.5, 0.02, and 0.15 μg/mL ABC, DTG, and 3TC, respectively, while high dose is composed of 4, 0.48, and 2.4 μg/mL ABC, DTG, and 3TC, respectively. Data are presented as the mean ± standard deviation of 3 independent experiments (biological replicates) each performed in triplicate (technical replicates). Data were analyzed with a one-way analysis of variance (ANOVA) followed by post-hoc comparisons, when appropriate. * p ≤ 0.05 ** p ≤ 0.01, *** p ≤ 0.001.


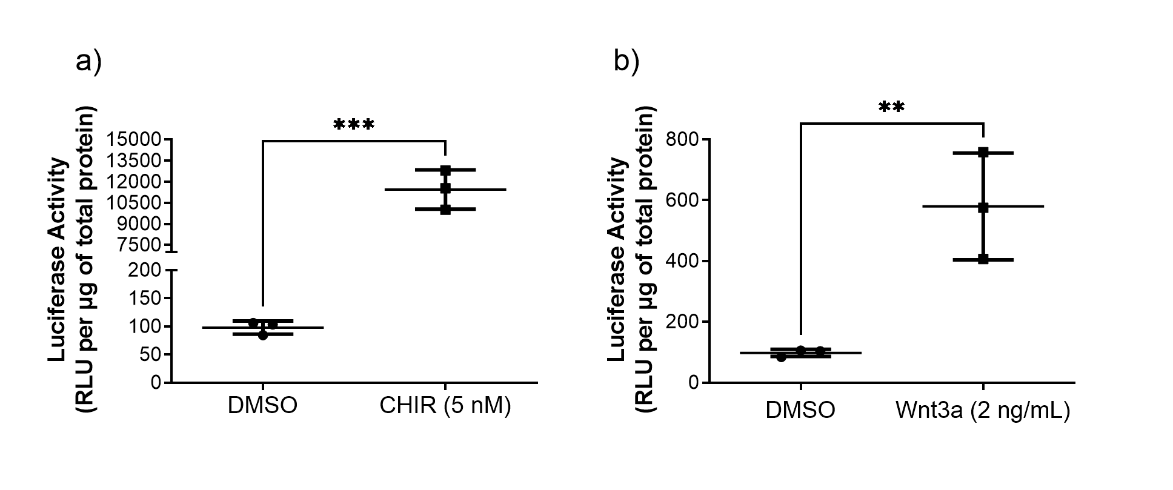


**Supplemental Figure** **3**: Wnt/β-catenin signaling pathway activation as measured by luciferase activity in TOPFlash transfected HOBs treated with **(a)** CHIR or **(b)** Wnt3a. Data are presented as mean ± standard deviation of 3 independent experiments (biological replicates) each performed in triplicate (technical replicates). Data were analyzed with a two-tailed T-test. * p ≤ 0.05 ** p ≤ 0.01, *** p ≤ 0.001


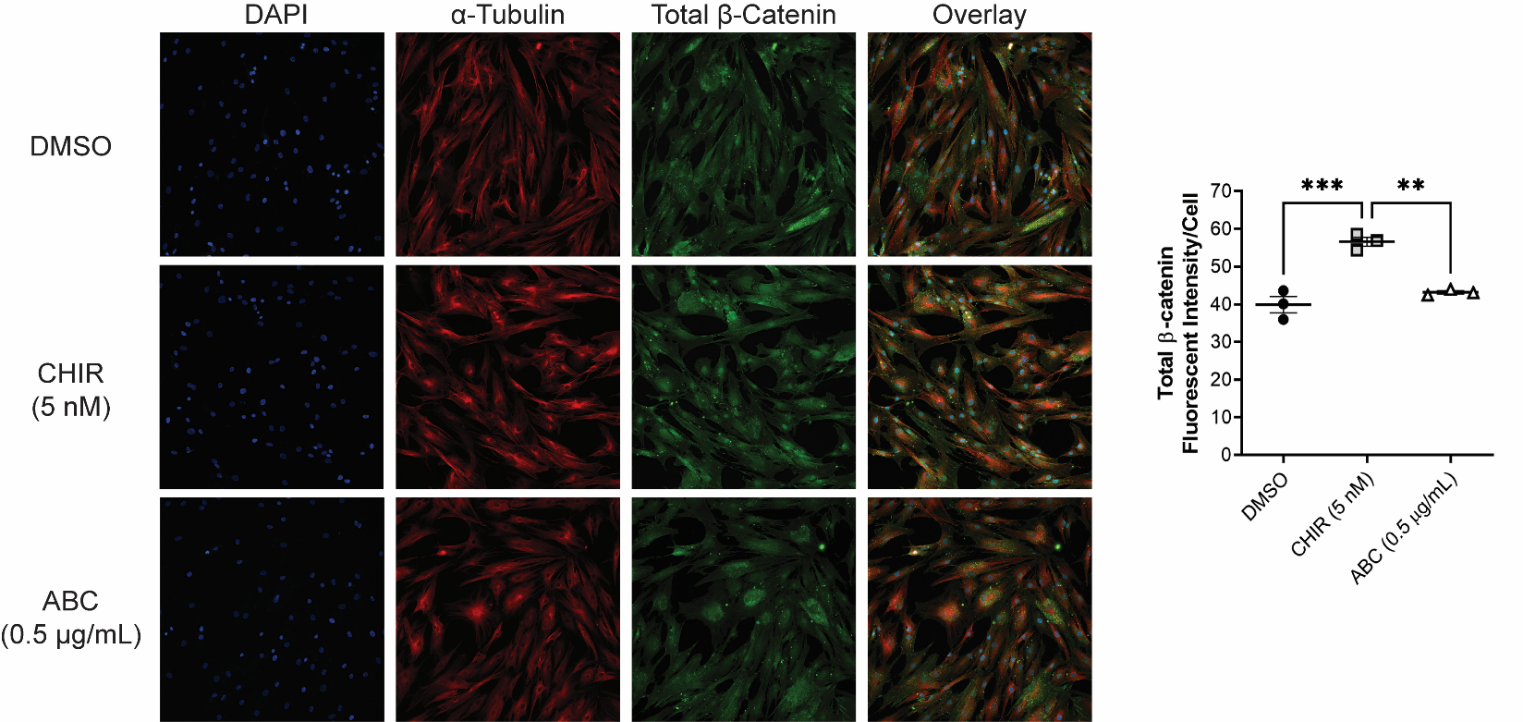


**Supplemental Figure 4:** Total β-catenin accumulation in primary human osteoblasts. Representative immunofluorescent images of DMSO (Control), CHIR, and ABC treated cells (left) and quantification of the signal intensity (right). The data is presented as mean ± standard deviation of 3 independent experiments (biological replicates). Data were analyzed with a one-way analysis of variance (ANOVA) followed by post-hoc comparisons, when appropriate. * p ≤ 0.05 ** p ≤ 0.01, *** p ≤ 0.001.


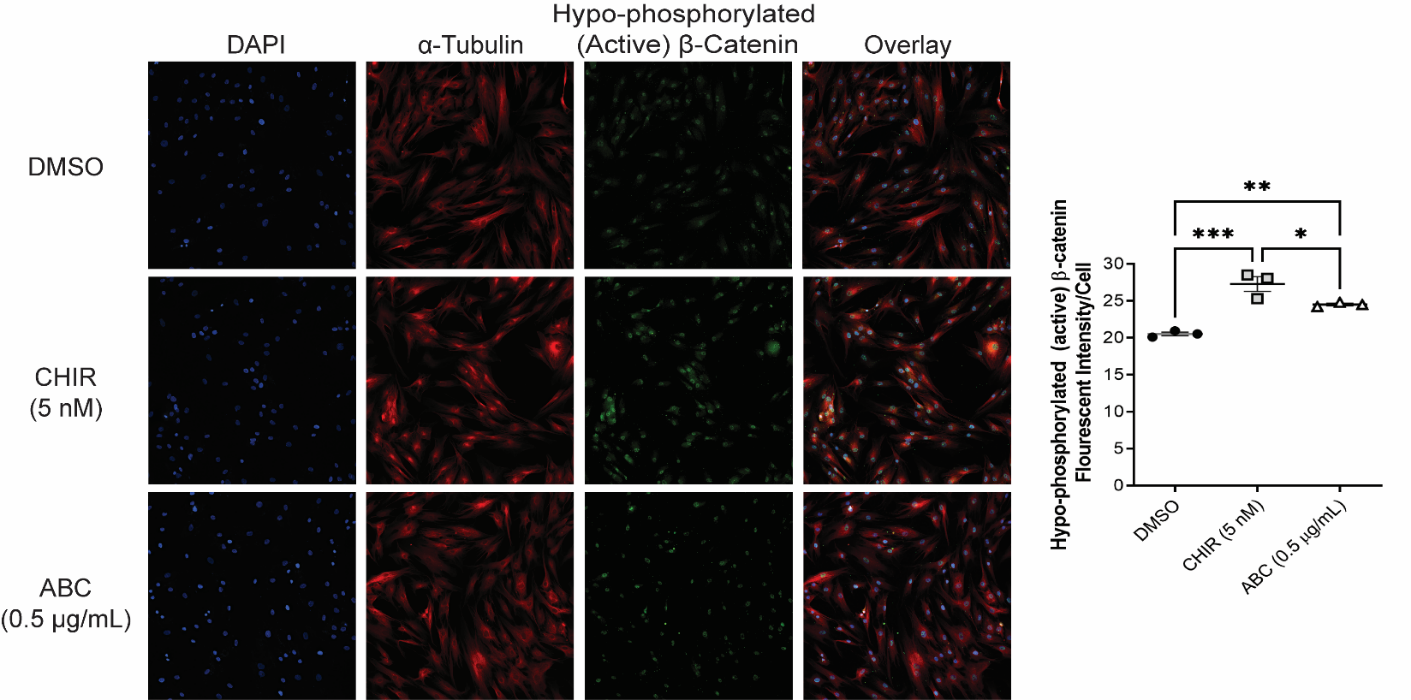


**Supplemental Figure 5:** Hypo-phosphorylated or active β-catenin accumulation in primary human osteoblasts. Representative immunofluorescent images of DMSO (Control), CHIR, and ABC treated cells (left) and quantification of the signal intensity (right). The data is presented as mean ± standard deviation of 3 independent experiments (biological replicates). Data were analyzed with a one-way analysis of variance (ANOVA) followed by post-hoc comparisons, when appropriate. * p ≤ 0.05 ** p ≤ 0.01.

**Supplemental Table 3:** Body weight, bone mineral density (BMD), bone structure, mechanics and turnover markers following 6-weeks of control or ABC treatment.

| **Parameter** | **Control** | **ABC/DTG/3TC** | **p-value** |
| --- | --- | --- | --- |
| Body weight (g) | 26.40 (1.95) | 24.60 (1.14) | 0.113 |
| Total body BMD (g/cm^2^) | 0.075 (0.002) | 0.076 (0.004) | 0.567 |
| Lumbar spine BMD (g/cm^2^) | 0.044 (0.004) | 0.044 (0.002) | 0.872 |
| Right femoral BMD (g/cm^2^) | 0.049 (0.004) | 0.049 (0.002) | 0.865 |
| Cortical Area (mm^2^) | 0.663 (0.103) | 0.618 (0.068) | 0.440 |
| Total Area (mm^2^) | 1.801 (0.161) | 1.670 (0.159) | 0.233 |
| Medullary Area (mm^2^) | 1.138 (0.072) | 1.052 (0.104) | 0.168 |
| Cortical Thickness (mm) | 0.128 (0.010) | 0.133 (0.010) | 0.447 |
| Cortical Porosity (%) | 1.221 (0.432) | 1.261 (0.197) | 0.853 |
| Peak Load (N) | 5.26 (2.44) | 5.45 (2.26) | 0.900 |
| Stiffness (N/mm^2^) | 35.96 (18.85) | 43.28 (16.59) | 0.532 |
| Femoral Bone volume fraction (BV/TV, %) | 11.43 (2.66) | 12.58 (2.05) | 0.466 |
| Femoral Trabecular Number (Tb.N, 1/mm) | 4.72 (0.25) | 5.08 (0.40) | 0.131 |
| Femoral Trabecular Thickness (Tb.Th, mm) | 0.045 (0.005) | 0.044 (0.004) | 0.563 |
| Femoral Trabecular Spacing (Tb.Sp, mm) | 0.209 (0.012) | 0.193 (0.017) | 0.109 |
| Femoral Connectivity Density (1/mm^3^) | 114.05 (24.15) | 146.81 (45.70) | 0.194 |
| Vertebral Bone volume fraction (BV/TV, %) | 23.27 (2.11) | 24.16 (2.38) | 0.547 |
| Vertebral Trabecular Number (Tb.N, 1/mm) | 5.65 (0.24) | 5.60 (0.44) | 0.822 |
| Vertebral Trabecular Thickness (Tb.Th, mm) | 0.046 (0.002) | 0.047 (0.001) | 0.184 |
| Vertebral Trabecular Spacing (Tb.Sp, mm) | 0.173 (0.008) | 0.177 (0.017) | 0.593 |
| Vertebral Connectivity Density (1/mm^3^) | 253.83 (22.15) | 226.53 (33.20) | 0.165 |
| The p-value presented is obtained from an unpaired students T-test. | | | |

1. Gregory CA, Gunn WG, Peister A, Prockop DJ. An Alizarin red-based assay of mineralization by adherent cells in culture: comparison with cetylpyridinium chloride extraction. Anal Biochem. 2004;329(1):77-84. doi:10.1016/j.ab.2004.02.002.
